# Sparse coding of pathology slides compared to transfer learning with deep neural networks

**DOI:** 10.1186/s12859-018-2504-8

**Published:** 2018-12-21

**Authors:** Will Fischer, Sanketh S. Moudgalya, Judith D. Cohn, Nga T. T. Nguyen, Garrett T. Kenyon

**Affiliations:** 1 Los Alamos National Laboratory, Los Alamos, NM USA; 2 Chester F. Carlson Center for Imaging Science, Rochester Institute of Technology, Rochester, NY USA

**Keywords:** Cancer pathology slides, TCGA, Sparse coding, Locally Competitive Algorithm, Unsupervised learning, Transfer learning, Deep learning

## Abstract

**Background:**

Histopathology images of tumor biopsies present unique challenges for applying machine learning to the diagnosis and treatment of cancer. The pathology slides are high resolution, often exceeding 1GB, have non-uniform dimensions, and often contain multiple tissue slices of varying sizes surrounded by large empty regions. The locations of abnormal or cancerous cells, which may constitute a small portion of any given tissue sample, are not annotated. Cancer image datasets are also extremely imbalanced, with most slides being associated with relatively common cancers. Since deep representations trained on natural photographs are unlikely to be optimal for classifying pathology slide images, which have different spectral ranges and spatial structure, we here describe an approach for learning features and inferring representations of cancer pathology slides based on sparse coding.

**Results:**

We show that conventional transfer learning using a state-of-the-art deep learning architecture pre-trained on ImageNet (RESNET) and fine tuned for a binary tumor/no-tumor classification task achieved between 85*%* and 86*%* accuracy. However, when all layers up to the last convolutional layer in RESNET are replaced with a single feature map inferred via a sparse coding using a dictionary optimized for sparse reconstruction of unlabeled pathology slides, classification performance improves to over 93*%*, corresponding to a 54*%* error reduction.

**Conclusions:**

We conclude that a feature dictionary optimized for biomedical imagery may in general support better classification performance than does conventional transfer learning using a dictionary pre-trained on natural images.

**Electronic supplementary material:**

The online version of this article (10.1186/s12859-018-2504-8) contains supplementary material, which is available to authorized users.

## Introduction

Images of tumor biopsies have a long history in oncology, and remain an important component of cancer diagnosis and treatment; they also provide promising opportunities for the application of machine learning to human health. Identifying the genetic signatures of cancer is an active area of research (reviewed in [1]); we examined a dataset [2] where genomic/transcriptomic data is augmented by high-magnification images of tissue samples. We hypothesize that the tissue images themselves might reveal tumor characteristics that would complement the information available in the associated gene expression data.

Medical imagery has been a target of artificial intelligence since the 1970s, and the majority of current approaches are based on “Deep Learning” using convolutional neural networks (reviewed in [3, 4]). Automated feature discovery has become increasingly common, and some have argued that “general purpose” image feature dictionaries (trained on ImageNet, for instance) may achieve high performance on specialized classification tasks [5–7]. Despite such reports of effective classification using features trained from conventional photographic databases, i.e., “transfer learning,” it remains unclear whether such features are truly optimal for the specialized task of tumor discrimination from cancer pathology slides, for which the low-level image statistics are likely to be very different.

Histological examination of tumor biopsies is a task currently performed by highly trained human pathologists, who assess the type and grade (progression stage) of tumors based on the appearance of thin tissue slices, typically stained with eosin and hematoxylin, in an optical microscope. In order to use machine learning to perform some of the tasks of a trained pathologist, we must first find representations of the pathology slides that display the most relevant information for characterizing tumors. Deep learning is an effective technique for learning representations, which yields good performance on a variety of classification tasks [8, 9]. However, conventional deep learning approaches are problematical here due to the large, non-uniform image sizes, limited amount of training examples and imbalanced nature of the image data, and the sometime necessity for labeling (e.g. annotations that distinguish normal from cancerous tissue within an image); much of the substantial body of work in this area has been focused on segmentation within an image [10] or limited to a small number of tumor types [7, 11–14].

Sparse coding has been shown to support near state-of-the-art performance on image labeling tasks using only a linear support vector machine (SVM) classifier [15, 16]. We hypothesize that sparse representations can similarly enable relatively shallow classifiers to achieve outstanding performance on the task of classifying pathology slides. While there have been some efforts to use sparse coding for classification of cancer pathology slides [10] to our knowledge no one has used dictionaries optimized for the sparse coding of cancer pathology slides in a transfer learning framework that exploits modern deep learning techniques. Our methodology comprises three steps:

1. Learn a dictionary via unsupervised optimization of sparse reconstruction using images drawn from a large training set;
2. Infer a sparse subset of nonzero feature activation coefficients for each image;
3. Classify the resulting sparse representations using a shallow neural network, or Multi-layer Perceptron (MLP).

Our methodology represents a form of transfer learning that covers many different tumor types and addresses the central histological classification problem: “Does the image on the slide contain cancerous tissue, or not?”

## Methods

### Image data

Image files for histologically stained micrographs of tumor slices were retrieved from the National Cancer Institute’s Genetic Data Commons (https://portal.gdc.cancer.gov/legacy-archive/search/f; as of September 2018, SVS images are available and can be viewed at https://portal.gdc.cancer.gov). Metadata for each image including ICD-10-CM codes [17] for both cancer type (morphology) and sample/biopsy anatomical location (topography) were retrieved from http://portal.gdc.cancer.gov. From 18,592 images associated with The Cancer Genome Atlas (TCGA) project, we selected a matched tumor/normal tissue subset, containing images from 691 distinct patients, with 1,375 distinct samples and 1,914 distinct histology image files. In each case, at least one image was available of normal tissue, and at least one image of tumor tissue from the same patient (derived from contemporaneous tissue-matched biopsies or distinct portions of the same biopsy). The final dataset included different slices from the same tumor, different tumor types from the same organ (e.g. breast, thyroid), and both similar and disparate tumor types from different tissues (Table 1; Additional file 1).

**Table 1:** Matched tumor/non-tumor tissue images

Tissue of origin	Tumor type	Count
Adrenal gland	Pheochromocytoma and Paraganglioma	6
Bile duct	Cholangiocarcinoma	18
Bladder	Bladder Urothelial Carcinoma	45
Breast	Breast Invasive Carcinoma	429
Colon	Colon Adenocarcinoma	130
Colon	Rectum Adenocarcinoma	27
Cervix	Cervical Squamous Cell Carcinoma and Endocervical Adenocarcinoma	6
Stomach	Stomach Adenocarcinoma	68
Head and neck	Head and Neck Squamous Cell Carcinoma	116
Lung	Lung Adenocarcinoma	179
Lung	Lung Squamous Cell Carcinoma	115
Liver	Liver Hepatocellular Carcinoma	118
Esophagus	Esophageal Carcinoma	16
Pancreas	Pancreatic Adenocarcinoma	8
Prostate	Prostate Adenocarcinoma	124
Kidney	Kidney Chromophobe	69
Kidney	Kidney Renal Clear Cell Carcinoma	214
Kidney	Kidney Renal Papillary Cell Carcinoma	78
Sarcoma	Sarcoma	4
Melanoma (skin)	Skin Cutaneous Melanoma	2
Thyroid	Thyroid Carcinoma	114
Thymus	Thymoma	4
Uterus	Uterine Corpus Endometrial Carcinoma	54

For each tumor from a given patient, at least one slide image was labeled as cancerous (“primary tumor”) and at least one image as “normal” (adjacent samples or clean margin)

### Image sectioning

**Fig. 1:**
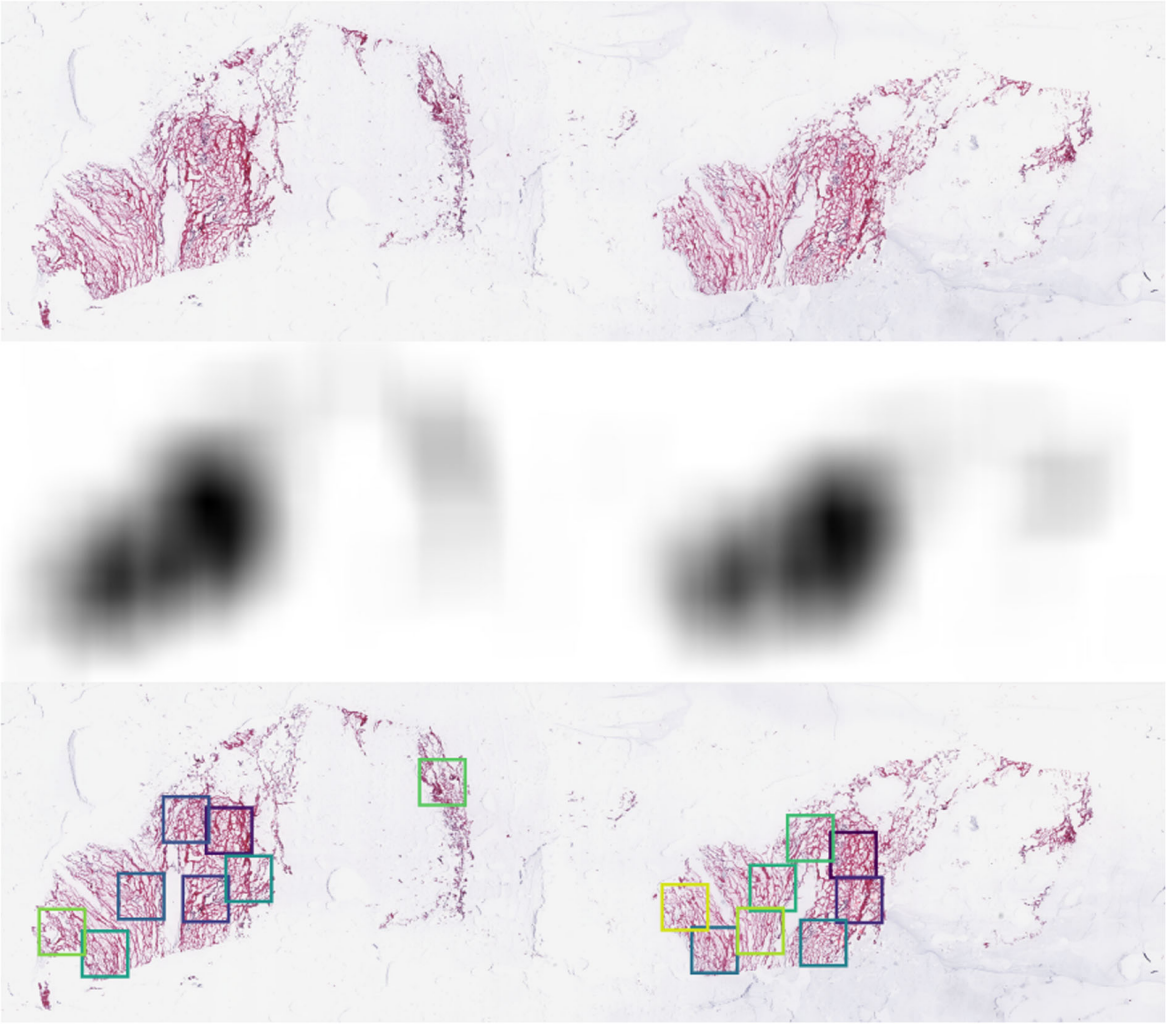
Preprocessing of TCGA pathology slides. Full-extent low-resolution images were used to determine image coordinates; full-resolution image slices were used to generate sparse representations. Top: initial image; center: fast Fourier transform versus all-white, to determine optically dark regions of the image; bottom: non-overlapping image slices representing a succession of darkest remaining portions of the image. Full resolution regions of interest (ROIs; colored boxes) were extracted from the SVS file; the four darkest ROIs from each image were used for the analyses reported here

**Fig. 2:**
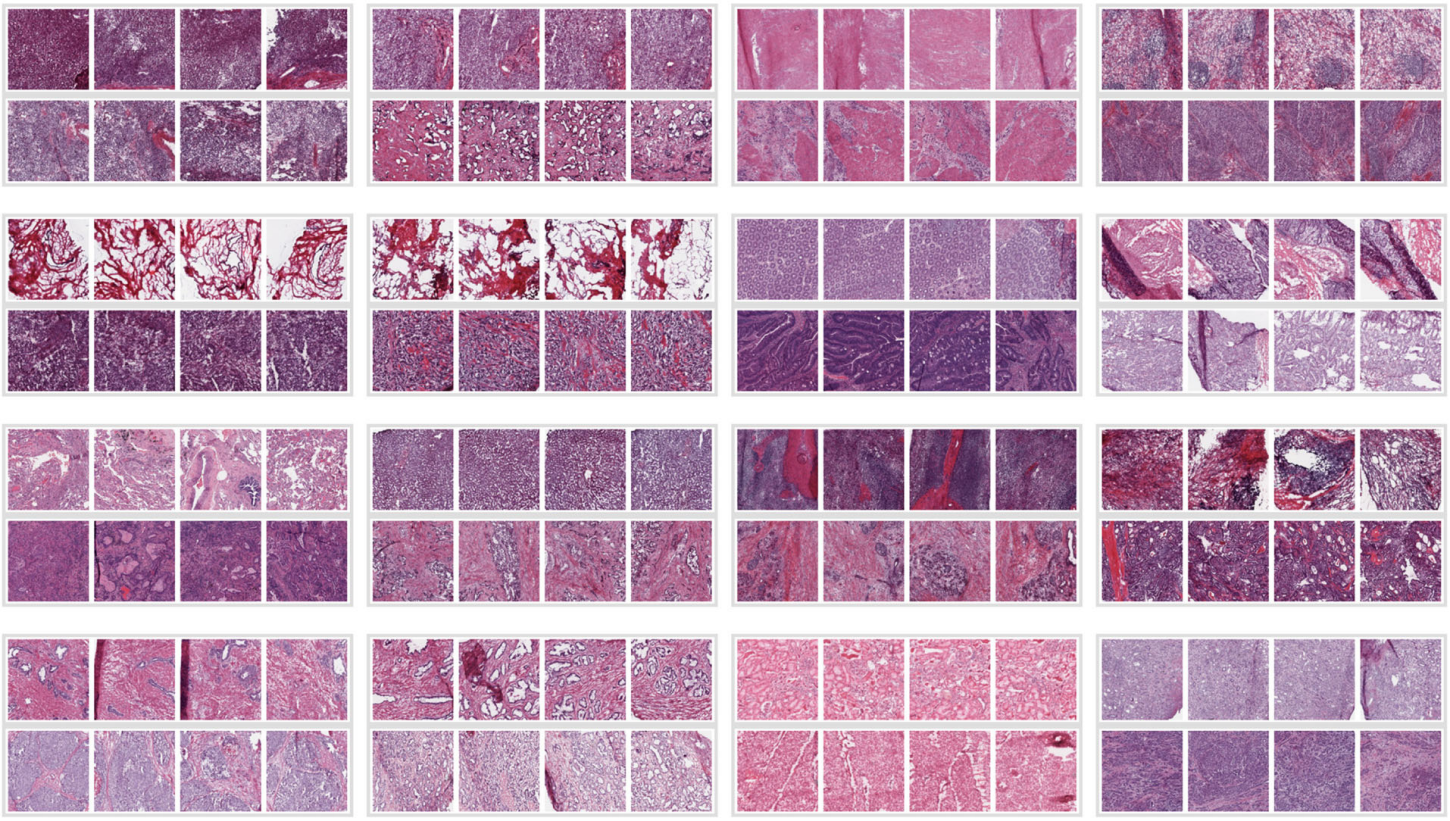
Sample region-of-interest (ROI) images. Each group of 8 small images contains ROIs derived from contemporaneous normal and tumor tissue samples from a single patient; within each group, the top row of 4 represents normal tissue; the bottom row, tumor tissue. Groups represent the following tumor types (left to right): row 1, adrenal, bile duct, bladder, stomach; row 2, breast, breast, colon, colon; row 3, lung, liver, pancreas, thyroid; row 4, prostate, prostate, kidney, kidney. Some sample pairs show overt tumor signatures (e.g., tissue disorganization, densely packed nuclei associated with rapid proliferation), but other samples lack such obvious features

Because individual slide images had large amounts of empty space, frequently presented multiple tissue slices on the same slide, and were of non-uniform size, we preprocessed each slide to extract several high-resolution samples. Regions of interest (ROIs) were selected by optical density. Starting with tiled SVS format image files, a variety of operations were performed using the openslide library [18], Octave [19], and custom Perl code. First, the lowest resolution available was extracted as a PNG format file; from this reference image, we extracted a number of non-overlapping square tiles of the desired size (2048×2048 pixels). Briefly, each image was binarized (using Otsu’s method [20] as applied in the “graythresh” function in Octave), and the white/non-white density was computed for each possible overlapping window using a fast Fourier transform (applying the fftconv2 function of the SPORCO library in Octave) [21]; the darkest non-overlapping sub-images were extracted sequentially. This simplistic heuristic ensured selection of non-empty regions, and favored densely staining regions. ROI coordinates defined on low-resolution images were used to extract the corresponding regions from the highest resolution images. These sub-images were scaled to yield the equivalent of 2048×2048 pixels at 20X magnification at full resolution. Figure 1 shows an example with 16 successive sub-samplings to illustrate the robustness of the procedure; for the work presented here, however, only the first four ROIs were used. Discrimination between matched tumor/non-tumor ROIs is non-trivial to the untrained eye (Fig. 2). Note that our method does not ensure that each ROI labeled as tumor contains cancer tissue, introducing some amount of noise in our training data.

### Sparse coding

Finding sparse representations of images is an important problem in computer vision, with applications including denoising, upsampling, compression [22, 23] and object detection [15, 16]. Moreover, sparse coding explains many of the response properties of simple cells in the mammalian primary visual cortex [24]. Given an overcomplete basis, sparse coding algorithms seek to identify the minimal set of generators that most accurately reconstruct each input image. In neural terms, each neuron is a generator that adds its associated feature vector to the reconstructed image with an amplitude equal to its activation. For any particular input image, the optimal sparse representation is given by the vector of neural activations that minimizes both image reconstruction error and the number of neurons with non-zero activity. Formally, finding a sparse representation involves finding the minimum of the following cost function:

1 $$ {}E\left(\overrightarrow{I}, \boldsymbol{\phi}, \overrightarrow{a}\right) = \min\limits_{\{\overrightarrow{a}, \, \phi \}} \left[ \frac{1}{2} \left\| \overrightarrow{I} - \boldsymbol{\phi} * \overrightarrow{a} \right\|^{2} \!+ \lambda \left\| \overrightarrow{a} \right\|_{1}.\right.   $$

In Eq. (1), $\overrightarrow {I}$ is an image unrolled into a vector, and ***ϕ*** is a dictionary of feature kernels that are convolved with the feature maps $\overrightarrow {a}$ that constitute a sparse representation of the image. The factor *λ* is a tradeoff parameter; larger *λ* values encourage greater sparsity (fewer non-zero coefficients) at the cost of greater reconstruction error.

Both the feature maps $\overrightarrow {a}$ and the dictionary of feature kernels ***ϕ*** can be determined by a variety of standard methods. Here, we solved for the feature maps using a convolutional generalization, previously described [16, 25], of the Locally Competitive Algorithm (LCA) [26], where the feature kernels themselves are adapted according to a local Hebbian learning rule that reduces reconstruction error given a sparse representation. Dictionary learning was thus performed via Stochastic Gradient Descent (SGD). Unsupervised dictionary learning used the entire data set. This was not perceived to be problematic as the learned features were clearly generic, and both tumor and non-tumor images were promiscuously intermingled. Both dictionary learning and sparse coding was performed using PetaVision [27], an open source neural simulation toolbox that uses MPI, OpenMP and CUDA libraries to enable multi-node, multi-core and/or GPU accelerated high-performance implementations of sparse solvers derived from LCA.

### Computing resources

All training was done on the Darwin cluster located at Los Alamos National Lab. Nodes used for both training and evaluation runs were typically configured with dual Intel Xeon CPUs with 40 virtual cores and single Nvidia graphic processors. Four nodes were used simultaneously: the GPUs were used to carry out non-sparse convolutions, while the CPUs were used for the sparse convolutions. This hybrid model, implemented using *openmpi*, *OpenMP*, and *cuDNN*, effectively utilized both CPU and GPU cores.

### Classification

After learning dictionaries, we inferred a sparse representation for each of 7,776 randomly ordered ROIs, 4,462 of which were drawn from slides labeled as containing tumor tissue. Although we drew 4 ROIs from each slide, we treated the (non-overlapping) ROIs as distinct samples. The feature maps for each ROI were average-pooled, producing a 512-element reduced representation of each ROI. The pooled representation for each ROI was used to train a linear support vector machine (SVM) [28] as well as an MLP to discriminate between ROIs derived from tumor and non-tumor slide images.

## Results

### Learned dictionary of convolutional feature kernels

**Fig. 3:**
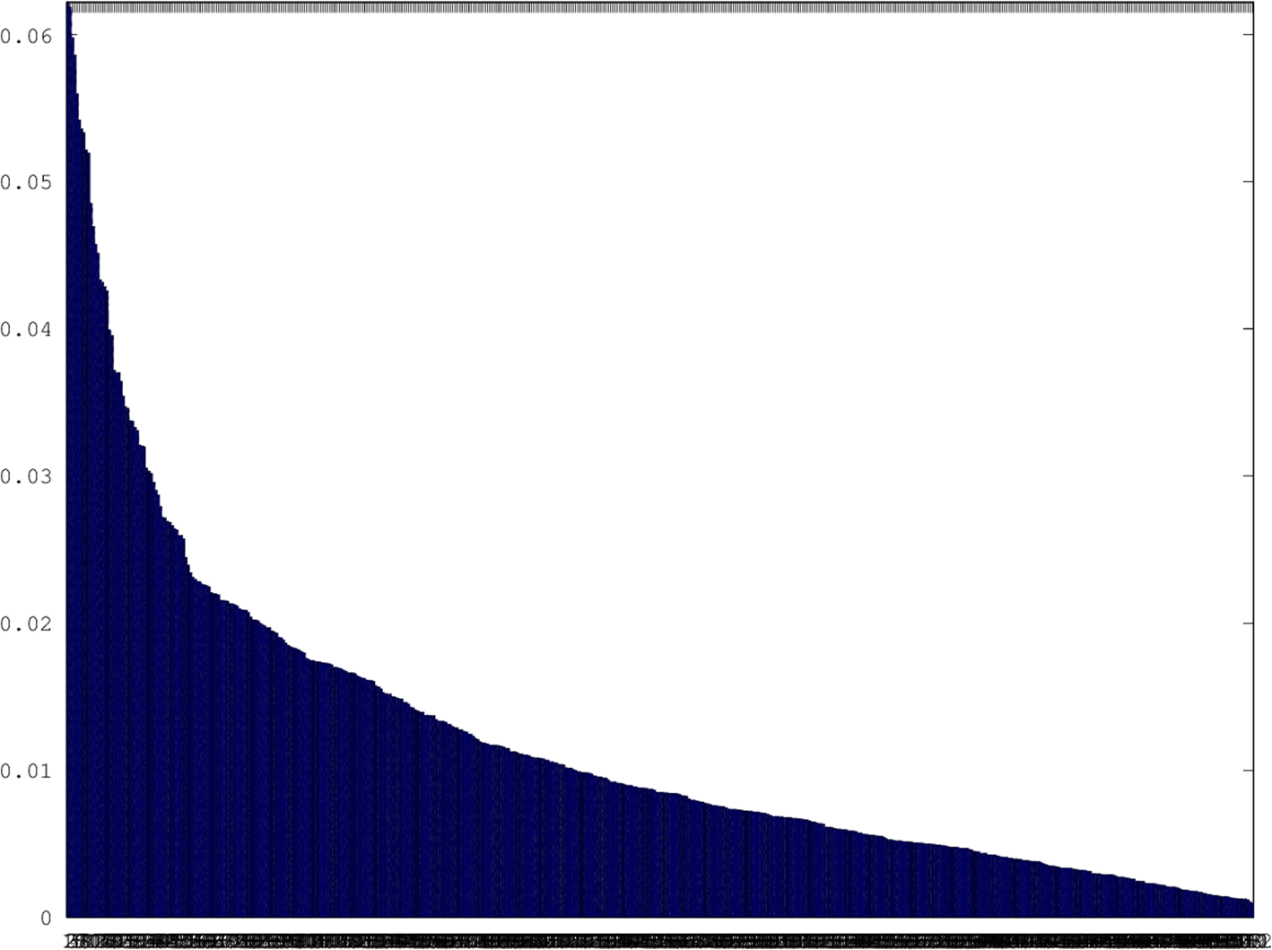
Distribution of feature coefficients. Histogram giving the percentage of non-zero activation coefficients for each of the 512 512×512 feature maps, averaged over a large set of ROIs

**Fig. 4:**
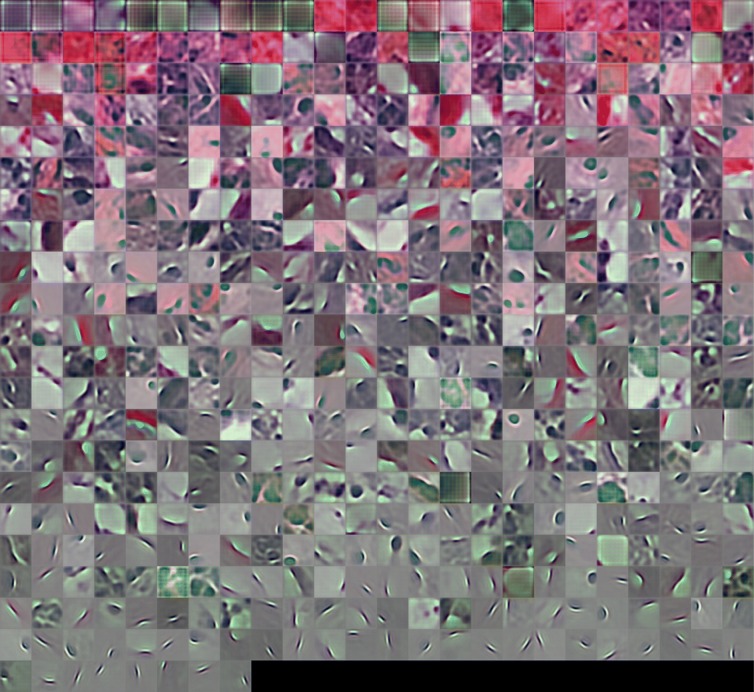
Feature dictionary. Dictionary of 512 convolutional feature kernels learned from the complete set of tumor and non-tumor image ROIs

We trained a convolutional dictionary for sparse reconstruction of 2048×2048 pixel full-resolution image slices (ROIs) extracted from TCGA images (Fig. 1). Each feature kernel was replicated with a stride of 4 pixels in both the vertical and horizontal directions, resulting in a feature map of size 512×512. The sparsity of the feature map is shown in Fig. 3. The set of 512 learned feature kernels can be visualized as RGB color image patches 32×32 in extent (Fig. 4). The learned dictionary is clearly specialized for pathology images. Although some feature kernels appear rather generic, representing short edge segments, typically with a slight curvature, many feature kernels resemble specific cytological structures. In particular, since the two different stains bind differentially to distinct cellular components (i.e., nucleic acid/chromatin vs protein/extracellular matrix), we expect feature kernels that combine spectral and structural elements to encode specific subcellular components. We hypothesize that some of the specialized feature kernels could be discriminative for tumor related pathologies.

### Image reconstructions

**Fig. 5:**
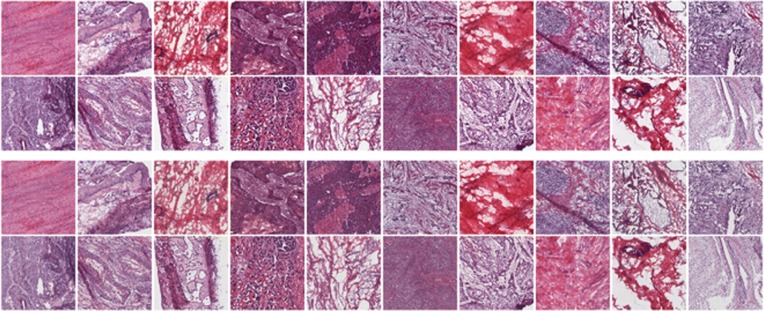
Image reconstructions. Samples of reconstructed images based on convolutional feature kernels and weights (coefficients). Top: original images; bottom: reconstructions

We evaluated the effectiveness of the image abstraction by reconstructing ROI images based on the feature dictionaries and the image-specific sparse coefficients. A sample of such reconstructions is shown in Fig. 5: although there are perceptible differences in color values, the reconstruction of fine structure is remarkably accurate.

### Discrimination between tumor/non-tumor

To test the hypothesis that sparse representations obtained using convolutional dictionaries optimized for the parsimonious representation of tumor images can be useful for classification, we used a linear support vector machine (SVM) [28] to perform binary discrimination of tumor versus non-tumor on each ROI. Input to the classifier consisted of the sparse feature maps, pooled to a 512-element vector corresponding to the average coefficient for each feature (average-pooling). By using a relatively simple linear SVM classifier, we were able to directly test the discriminative power of the sparse representations themselves without the confound of additional nonlinearities. The classification accuracy we achieved (84.23*%*, with chance performance of 56*%* due to the slight preponderance of tumor slices in the dataset) shows that our unsupervised sparse representations captured some aspects of tumorous versus non-tumorous tissue – i.e., some generic features such as (possibly) a preponderance of proliferating nuclei. We also tried max-pooling and histogramming activation coefficients but obtained poorer classification results (data not shown).

### Transfer learning based on sparse coding

As a control, we employed a state-of-the-art deep learning architecture for image classification, Residual Network (RESNET), to examine performance of conventional transfer learning on our dataset. We started with RESNET-152 from Keras libraries built in TensorFlow using previously learned weights [29, 30], obtained from about a million training images [31]. We retrained the final all-to-all layers from scratch on the same TCGA ROI images as used above. The convolutional layers were fine-tuned as well. The first all-to-all layer consisted of 1,000 fully-connected elements followed by a drop-out and a softmax layer. Thus, we began with convolutional features optimized for classifying natural images but used the available training data to adapt an existing RESNET architecture for classifying cancer pathology slides. Training/test subsets were approximately in the ratio of 5/1, respectively. We obtained a classification score of 85.48*%*±0.36*%* on holdout test data, slightly higher than our score obtained by feeding sparse coefficients into a linear SVM classifier (84.32*%*).

Next, we employed an analogous transfer learning approach using our sparse coding feature map fed directly into the all-to-all layers at the top of the RESNET architecture. These all-to-all layers consisted of a fully-connected 512-element table, a drop-out layer, and a softmax classification layer. Again, training/test subsets were approximately in the ratio of 5/1. For the transfer learning approach based on sparse coding, we obtained a classification accuracy of 93.32*%*±0.21*%*, approximately 54*%* error reduction from the conventional transfer learning approach. Classification performance of the 3 approaches is shown in Table 2.

**Table 2:** Summary of classification performances

Approach	Classification score
Sparse coding, SVM	84.23%
RESNET-152	85.48 ±0.36%
Sparse coding, MLP	93.32 ±0.21%

## Discussion

Our results suggest that optimizing a dictionary for a sparse coding directly on raw unlabeled histological data and using that dictionary to infer sparse representations on each image can support substantially better performance than transfer learning based on features optimized for natural images [5]. An approach based on sparse coding yields features specialized for the parsimonious reconstruction of histology slides, without requiring either extensive hand-labeling or segmentation of images, and yet achieves respectable classification accuracy. The fact that features learned in an unsupervised manner can nonetheless support accurate classification might at first seem surprising. State-of-the-art deep neural networks, trained in a fully supervised manner so as to yield a maximally discriminative set of features, approach human levels of performance on a variety of benchmark image classification tasks. Features trained in an unsupervised manner for sparse reconstruction, on the other hand, are not required to be discriminative *per se* (e.g. between cancerous and non-cancerous tissue), but *are* required to enable parsimonious descriptions of the data. In the case of histology slides, it is not unreasonable that features optimized for sparse reconstruction might naturally correspond to physiologically meaningful entities, such as cell membrane, cytoplasm, nuclear material and other subcellular structures, as such features likely enable the most parsimonious explanation of the data. Occasionally, such physiologically-meaningful features will be naturally discriminative between cancerous and non-cancerous tissue even though such discrimination was not explicitly optimized for. While deep learning approaches would likely have produced superior results *given enough labeled training examples*, such labeled datasets can only be prepared by highly trained pathologists and are currently unavailable. Instead, we started with a deep neural network optimized for the classification of natural images, which are clearly very different from pathology slides, and would be unlikely to contain features corresponding to subcellular components. Absent sufficient labeled training data, our results indicate that a hybrid approach based on unsupervised sparse coding followed by a relatively shallow but non-linear fully-supervised classifier supports the best classification performance. Finally, we attempted no systematic search of meta-parameters to optimize the classification performance supported by our hybrid approach based on sparse coding followed by an MLP with a single hidden layer. Thus, it is likely that our reported classification performance could be improved by optimizing various meta-parameters such as the patch size, number of dictionary elements and overall sparsity [32].

## Conclusions

The results reported here provide a proof-of-concept for discrimination between cancer and non-cancer by sparse coding of histopathological images fed into a shallow three-layer neural net (MLP). High classification accuracy was achieved even though features were learned without labeling (i.e. with no reference to the presence or absence of tumor within any given ROI). These results indicate that a subset of sparse feature kernels generated by unsupervised training can be discriminative between tumor and non-tumor.

Although some researchers have used transfer learning to compensate for a limited number of training examples, it is unclear whether features optimized for natural images will support high levels of classification performance on cancer pathology slides, even after fine tuning on the target data. Here, we report that sparse feature encoding on unlabeled target data substantially improves performance.

## Additional file

Additional file 1 Tab-delimited file
1. tcga_hist_file_name (original name of image file as downloaded from Genomic Data Commons)
2. tcga_project_code
3. tumor_type (TCGA project tumor type)
4. iocd_topo_code (IOCD topographical code for tumor sample)*
5. iocd_morph_code (IOCD morphological code for tumor sample)*
6. patient_id (TCGA patient ID)
7. sample_id (TCGA sample ID)
8. sample_type (Primary Tumor, Solid Tissue Normal, or Metastatic)
* normal samples are taken from the vicinity of tumor samples and are labelled with the same IOCD codes. (TXT 294 kb)
